# Digital study and correlation analysis of sagittal balance parameters in adolescents with idiopathic scoliosis: A comparative study with healthy adolescents

**DOI:** 10.1371/journal.pone.0326233

**Published:** 2025-06-18

**Authors:** Zhijie Kang, Guopeng Shi, Yunfeng Zhang, Feng Jin, Kai Zhang, Zhenhua Cao, Yong Zhu, Yangyang Xu, Yuan Fang, Lirong Sha, Bin Liu, Qi Yang, Yingying Jiang, Haiyan Wang, Xiaohe Li

**Affiliations:** 1 Department of Human Anatomy, School of Basic Medicine, Inner Mongolia Medical University, Hohhot, Inner Mongolia, China; 2 Department of Radiology, Second Affiliated Hospital, Inner Mongolia Medical University, Hohhot, Inner Mongolia, China; 3 Department of Radiology, Affiliated Hospital, Inner Mongolia Medical University, Hohhot, Inner Mongolia, China; 4 Orthopedics, Second Hospital of Ulanqab City, Ulanqab, Inner Mongolia, China; 5 Third Hospital, Jinzhou Medical University, Liaoning Province, China; 6 Tumor Hospital, affiliated to Inner Mongolia Medical University, Inner Mongolia Medical University, Hohhot, Inner Mongolia, China; 7 Rehabilitation Medicine Department, People’s Hospital of Changzhi City, Shanxi Province, China; 8 Clinical Medical College, Baotou, Inner Mongolia Medical University, Hohhot, Inner Mongolia, China; 9 Clinical Medical College, Tongliao, Inner Mongolia Medical University, Hohhot, Inner Mongolia, China; 10 Clinical Medical College, Bayan Nur, Inner Mongolia Medical University, Hohhot, Inner Mongolia, China; Ningbo University, CHINA

## Abstract

**Purpose:**

This study aimed to compare sagittal spinal parameters between healthy adolescents and those with adolescent idiopathic scoliosis (AIS), identify factors influencing disease progression, and provide insights for optimizing preoperative assessments.

**Methods:**

Sagittal full-spine radiographs from 40 healthy adolescents and 41 AIS patients (aged 10–18 years) were analyzed using Mimics 21.0 software. Fifteen parameters, including spinopelvic angle (SPA), thoracic kyphosis (TK), lumbar lordosis (LL), and pelvic tilt (PT), were measured. Statistical analyses included logistic regression to identify predictors of AIS and sex-based subgroup comparisons.

**Results:**

SPA was significantly higher in AIS patients compared to controls (median: 176.48° vs. 169.64°, P = 0.008) and emerged as the sole predictor of AIS (odds ratio = 1.568, 95% CI = 1.129–2.177, P = 0.007). Sex differences revealed higher spinal tilt (ST) in female AIS patients (P = 0.034), while males exhibited elevated TK (P = 0.006) and SPA (P = 0.002). Correlations among parameters highlighted strong associations between LL and pelvic incidence (PI, r = 0.682) and between SPA and pelvic tilt (PT, r=−0.537).

**Conclusion:**

Increased SPA is a critical indicator of AIS, necessitating preoperative evaluation of sagittal spinopelvic alignment. Female patients require heightened attention to spinal tilt. Future studies should expand sample sizes and integrate multi-planar analyses to refine clinical strategies.

## Introduction

Adolescent idiopathic scoliosis (AIS) is a condition characterized by an unexplained lateral curvature of the spine that develops during adolescence, leading to a three-dimensional deformity of the spine [1]. As the condition progresses, spinal curvature manifests as an asymmetrical deformity that affects the physiological curvature of the spine and morphology of the pelvis [2]. However, early in puberty, AIS often presents with no obvious clinical symptoms, making it easy to overlook [3]. If not diagnosed and treated in a timely manner, this condition can lead to progressive spinal curvature, severely affecting the morphological and functional body structures of adolescents [4]. Moreover, patients with AIS may experience psychological issues, such as depression, anxiety, and mental disorders, owing to changes in appearance, discomfort, and treatment stress, adding to the burden on families and society [1]. The preferred method for diagnosing AIS is radiography with a Cobb angle of ≥10° in the coronal standing position [5]. Nonsurgical treatments are adopted for patients with mild-to-moderate AIS, whereas surgical treatments are used in severe cases [6].

A normal spine exhibits physiological curvatures in the sagittal plane, including cervical, thoracic, lumbar, and sacral curvatures, which are key to maintaining an upright posture, buffering stress from movement, and physiological activities [7,8]. When scoliotic deformities occur, vertebral rotation can cause a shift in the coronal plane; in severe cases, a noticeable sagittal imbalance may arise [9]. When the overall sagittal balance of the spine is disrupted, patients can experience a decrease in the quality of life and spinal functional impairments. The spinal deformities and rotation in patients with AIS can lead to changes in their original physiological curves. The affected segments of the spine deviate from their original trajectory, resulting in a loss of the spine’s biomechanical characteristics, This alteration also changes the body’s posture and gait patterns, potentially leading to slower walking speeds and asymmetries in both horizontal and vertical ground reaction forces [10,11]. Therefore, before treating the disease, physicians need to fully understand the patient’s sagittal spinal alignment to formulate a more accurate treatment plan [12–14]. During the early treatment of AIS, doctors often focus excessively on correcting coronal deformities, neglecting the importance of correcting sagittal balance [15]. Reconstructing sagittal deformities can improve patient’s quality of life and is the key to extending patient prognosis [16,17].

To date, although there have been studies on the sagittal spinal and pelvic parameters of patients with AIS, research exploring the differences in sagittal balance parameters between healthy adolescents and patients with AIS is limited and restricted [16,18]. Therefore, this study collected sagittal full-spine X-ray imaging data from 40 healthy adolescents and 41 patients with AIS and compared 15 sagittal parameters that can assess the overall spinal status to analyze their differences and clinical significance.

## Materials and methods

### 2.1 Data collection

**Fig 1:**
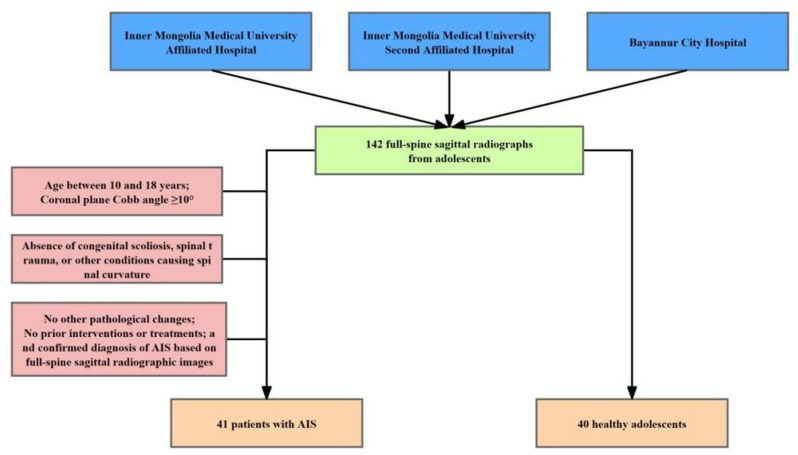
Schematic diagram of data sources.

This study collected sagittal full-spine radiographs of adolescents aged 10–18 years from January 2022 to May 2023, obtaining 142 radiographic records. These data were sourced from the Affiliated Hospital of Inner Mongolia Medical University, Second Affiliated Hospital of Inner Mongolia Medical University, and Bayannur City Hospital. After screening, 81 participants were included in the study, comprising 40 healthy adolescents and 41 patients with AIS (**Fig 1**).

### 2.2 Inclusion and exclusion criteria

Criteria for healthy adolescents: Normal bone quality in the cervical, thoracic, lumbar spine, and pelvis, with normal curvature in the thoracic and lumbar regions, intact vertebral bone structure, no narrowing of the intervertebral spaces, no scoliosis, and no other spinal deformities, determined to be healthy adolescents based on clinical assessment.

Criteria for patients with AIS: Aged between 10–18 years, with a Cobb angle of ≥10° in the coronal position, no congenital scoliosis, no spinal trauma or scoliosis caused by other diseases, no other pathological changes, have not received any intervention treatments, and confirmed as patients with AIS having full-spine sagittal X-ray images.

### 2.3 Research methodology

#### 2.3.1 Instrumentation and projection parameters.

X-ray imaging of the adolescent full-spine sagittal view was performed using Philips DR (DigitalDiagnost, Netherlands) and Siemens DR (Multix Fusion, Germany). Prior to imaging, subjects were instructed to stand with their faces towards the plate, eyes gazing forward, hips and knees extended, feet shoulder-width apart, and arms flexed at 90° and held horizontally in front of the chest to ensure high-quality X-ray images. The exposure distance was set at 200 cm, with a tube voltage of 120 kV and a tube current of 60 mA. All subjects were positioned uniformly to ensure consistency in data acquisition.

#### 2.3.2 Parameter measurements.

The scanned sagittal full spine X-ray raw image data were imported into Mimics 21.0 (License No: 9AF5–7882-B4DF-F7F3), in the DICOM format. “New Project” in “File” was selected to open the data and determine the orientation, and the coronal position was displayed after success. In “Measure,” “Angle” was selected to measure the angle; “Distance” was selected to measure the required distance. After the measurement was completed, the measurement result in “measurement” was selected and then “Export” was selected to export, which was convenient for subsequent organization and statistics.

#### 2.3.3 Methods for measuring sagittal balance parameters.

**Fig 2:**
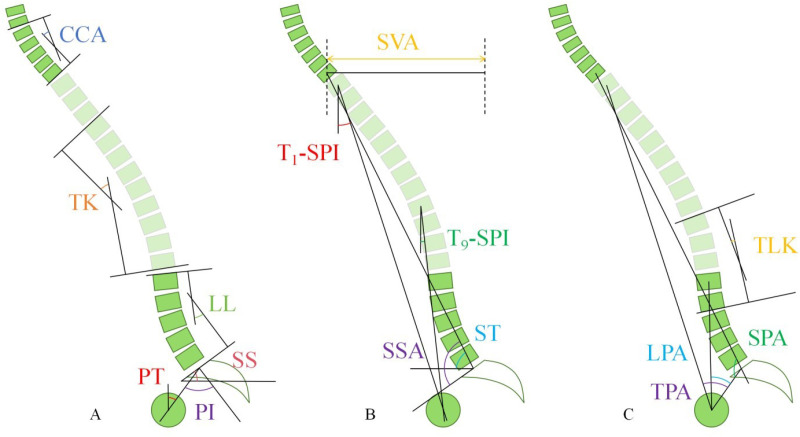
Schematic of the sagittal plane parameter measurements [21].

The sagittal balance parameters were measured as follows:

1. Cervical Cobb angle(CCA): angle between the lower endplates of C2 and C7 (**Fig 2A-CCA**) [17].
2. Thoracic kyphosis (TK): The Cobb angle between the upper endplate of T4 and the lower endplate of T12 (**Fig 2A-TK**) [9].
3. Lumbar lordosis (LL): Cobb angle between the upper endplates of L1 and S1 (**Fig 2A-LL**) [9].
4. Thoracolumbar kyphosis(TLK): Cobb angle between the upper endplate of T10 and the lower endplate of L2 (**Fig 2C-TLK**) [9].
5. Sagittal vertical axis (SVA): The horizontal distance between the vertical line through the center of C7 and the posterior superior corner of S1, with a positive value if the plumb line is anterior to the superior posterior edge of S1 and a negative value if it is posterior (**Fig 2B-SVA**) [7].
6. Spinal tilt (ST): angle between the line connecting the midpoint of C7 to the midpoint of the upper endplate of S1 and the horizontal line(**Fig 2B-ST**) [19].
7. Spinosacral angle (SSA): angle between the line connecting the center of C7 to the midpoint of the upper endplate of S1 and the upper endplate of S1 (**Fig 2B-SSA**) [19].
8. Spinopelvic angle (SPA): The angle made by the line connecting the midpoint of the upper endplate of S1 with the midpoints of C7 and the femoral head (**Fig 2C-SPA**) [20].
9. T1 spinopelvic inclination (T1-SPI): The angle between the line from the midpoint of T1 to the midpoint of the femoral head and a vertical line through the midpoint of T1, with a positive value if the line inclines forward relative to the vertical line and a negative value if it inclines backward (**Fig 2B- T**_**1**_**-SPI**) [18].
10. T9 spinopelvic inclination (T9-SPI): The angle between the line from the midpoint of T9 to the midpoint of the femoral heads and a vertical line through the midpoint of T9, with a positive value if the line inclines forward relative to the vertical line and a negative value if it inclines backward (**Fig 2B- T**_**9**_**-SPI**) [18].
11. T1 pelvic angle(TPA): The angle formed by the line connecting the midpoint of the femoral head with the midpoint of the upper endplate of S1 and the midpoint of T1 (**Fig 2C- TPA**) [18].
12. Lumbar pelvic angle(LPA): the angle formed by the line connecting the midpoint of the femoral heads to the midpoint of L1 and the midpoint of the upper endplate of S1 (**Fig 2C- LPA**) [18].
13. Sacral slope (SS): The angle between the upper edge of S1 and the horizontal line (**Fig 2A-SS**) [9].
14. Pelvic incidence (PI): The angle between the line from the midpoint of the upper edge of S1 to the center point of the femoral head and a vertical line through the midpoint of the upper edge of S1 (**Fig 2A-PI**) [9].
15. Pelvic tilt (PT): The angle between the line from the midpoint of the upper edge of S1 to the center point of the femoral heads and the vertical line (**Fig 2A-PT**) [9].

#### 2.3.4 Measurement program.

All sagittal parameters were measured using repeated measurements by multiple individuals. Standardized training was used to determine the measurement protocol before measurement. The doctors were divided into two groups, A and B. Each group consisted of one chief spine surgeon and one chief imaging physician, and each doctor measured all the sagittal parameters. After the first measurement, the imaging data were disordered, and the four doctors were allowed to repeat the measurements two weeks later to find the average of the two measurements of each person. If the difference in the measurement results was too large when the data were summed, the results were determined by four doctors after consultation.

#### 2.3.5 Relevance criteria.

Judgment criteria of correlation coefficient: 1) complete correlation when |r| = 1; 2) weak correlation or slight correlation when 0 < |r| ≤ 0.4; 3) moderate correlation when 0.4 < |r| ≤ 0.6; 4) significant correlation when 0.6 < |r| ≤ 0.8; and 5) high correlation when 0.8 < |r| < 1. [22]

#### 2.3.6 Research statement.

All methods were performed in accordance with the relevant guidelines and regulations. The study protocol was approved (YKD2019138) by the Ethics Committee of Inner Mongolia Medical University.

### 2.4 Statistical analysis

The results of the measurement data were entered into SPSS (version 26.0; SPSS Inc., USA) for statistical analysis to compare the differences in the aforementioned parameters, and all the measurement parameters were analyzed for correlations. When the data conformed to normal distribution and the chi-square test, the measurement data were expressed as one-way ANOVA, and the LSD t-test was used for the two-way test of multiple independent samples. When the data did not conform to normal distribution and chi-square, the measurement data were expressed as the median (lower quartile and upper quartile). The Kruskal–Wallis H test was used, and the Nemenyi test was used for the two-way comparison of multiple independent samples. Count data were expressed as cases, and the Nemenyi test was used for two-way comparisons of multiple independent samples. The count data were expressed as the number of cases and percentages; the correlation analysis between each parameter was performed by Pearson/Spearman correlation analysis; binary logistic regression was used to analyze the relation between the different sagittal parameters and the disease, and *P < *0.05 was statistically significant.

## Results

### 3.1 General description of the data

This study included 81 participants with complete adolescent full-spine sagittal X-ray images, consisting of 40 healthy adolescents and 41 adolescent patients with AIS. The 40 healthy adolescents included 19 men and 21 women, with an average age of 14.73 years (range: 11–18 years). The 41 patients with AIS included 20 men and 21 women, with an average age of 14.80 years (range: 10–18 years). There were 65 curves in patients with AIS, with an average Cobb angle of 23.50° (range 14°–48°), including 18 patients with mild scoliosis (10°–19°), 21 with moderate scoliosis (20°–39°), and two with severe scoliosis (40°and above).

### 3.2 Sagittal parameters in healthy adolescents and patients with AIS

The analysis of sagittal parameters between healthy adolescents and patients with AIS showed that the SPA was statistically significant (*Z = *−2.636, *P = *0.008), whereas the rest showed no statistical significance (*P > *0.05) (Table 1).

**Table 1:** Comparison of sagittal parameters between healthy adolescents and patients with AIS[$\overset{―}{x}$±SD/M (Q_L_,Q_U_)].

Sagittal Parameters	Healthy Adolescents	AIS	*t/Z*	*p*
TK(°)	29.10 ± 7.27	31.56 ± 7.48	2.246	0.138
LL(°)	48.13 ± 9.47	49.17 ± 9.14	0.254	0.616
PT(°)	11.30(5.16-14.03)	7.67 (3.23-12.74)	−1.143	0.253
SS(°)	36.34 ± 8.43	36.60 ± 7.70	0.020	0.887
PI(°)	46.58 ± 9.67	45.01 ± 9.32	0.551	0.460
CCA(°)	10.28(6.50-16.97)	12.96(10.20-17.45)	−1.743	0.081
TLK(°)	11.87(9.26-15.30)	11.85 (8.71-16.24)	−0.019	0.985
SVA(mm)	6.88 ± 32.23	2.55 ± 28.57	0.411	0.523
SSA(°)	125.99 ± 8.05	126.42 ± 6.71	0.069	0.793
ST(°)	89.85 ± 3.49	89.56 ± 4.32	0.112	0.739
SPA(°)	169.64(165.84-176.64)	176.48(170.42-178.09)	−2.636	0.008
T1-SPI(°)	−3.01(−4.68--1.45)	−1.97(−3.67--0.74)	−1.091	0.275
T9-SPI(°)	−7.39(−9.41--5.64)	−7.79 (−8.85--3.34)	−0.836	0.403
TPA(°)	7.80(1.90-10.88)	7.50(5.04-11.48)	−0.916	0.359
LPA(°)	5.00 (2.28-7.80)	6.33(4.39-9.08)	−1.852	0.064

### 3.3 Sex analysis in patients with AIS.

In patients with AIS, significant statistical differences were observed between sexes in TK, PT, SS, ST, SPA, T1-SPI, and T9-SPI (*t = *8.363, *P = *0.006; *Z = *−3.717, *P = *0.000; *t = *5.675, *P = *0.022; *t = *4.823, *P = *0.034; *Z = *−3.078, *P = *0.002; *Z = *−2.556, *P = *0.011; *Z = *−2.269, *P = *0.023); no significant statistical differences were found in the remaining parameters (*P > *0.05) (Table 2).

**Table 2:** Comparison of parameters between sexes in patients with AIS.

Sagittal Parameters	male	female	*t/Z*	*p*
TK(°)	34.74 ± 8.00	28.53 ± 5.60	8.363	0.006
LL(°)	51.52 ± 8.94	46.94 ± 8.96	2.691	0.109
PT(°)	3.72(2.70-7.69)	12.05(7.04-17.00)	−3.717	0.000
SS(°)	39.38 ± 6.29	33.95 ± 8.12	5.675	0.022
PI(°)	44.52 ± 8.23	45.48 ± 10.43	0.105	0.747
CCA(°)	12.67 (7.18-19.16)	13.10(10.87-17.03)	−0.730	0.465
TLK(°)	11.68(8.93-16.09)	13.28 (8.51-18.13)	−0.078	0.938
SVA(mm)	8.78(−7.42-28.77)	3.23(−14.71-16.98)	−0.678	0.498
SSA(°)	127.99 ± 6.13	124.92 ± 7.04	2.202	0.146
ST(°)	88.11 ± 3.54	90.94 ± 4.61	4.823	0.034
SPA(°)	177.97(176.23-178.77)	171.42(167.40-176.70)	−3.078	0.002
T1-SPI(°)	−1.68(−3.03-2.62)	−2.88(−6.70--1.90)	−2.556	0.011
T9-SPI(°)	−4.19(−8.12--3.19)	−8.49(−11.92--4.45)	−2.269	0.023
TPA(°)	7.68 ± 5.67	9.24 ± 5.39	0.814	0.372
LPA(°)	6.01(3.85-9.28)	6.34 (5.01-9.08)	−0.756	0.449

[$\overset{―}{x}$±SD/M(Q_L_,Q_U_]

### 3.4 Correlation analysis of sagittal plane parameters in patients with AIS

LL showed a weak positive correlation with TK, and significant positive correlations with SS, PI, and SSA (*r = *0.335, *P = *0.033; *r = *0.776, *P = *0.000; *r = *0.682, *P = *0.000; *r = *0.762, *P = *0.000); PT showed a weak positive correlation with PI and ST, a moderate negative correlation with SPA and T1-SPI, and a significant negative correlation with T9-SPI (*r = *0.370, *P = *0.017; *r = *0.335, *P = *0.032; *r = *−0.537, *P = *0.000; *r = *−0.579, *P = *0.000; *r = *−0.639, *P = *0.000); SS showed a significant positive correlation with PI and a high positive correlation with SSA, a weak positive correlation with T9-SPI, and a weak negative correlation with CCA(*r = *0.731, *P = *0.000; *r = *0.815, *P = *0.000; *r = *0.352, *P = *0.024; *r = *−0.312, *P = *0.047); PI showed a significant positive correlation with SSA and a moderate negative correlation with SPA (*r = *0.664, *P = *0.000; *r = *−0.417, *P = *0.007); ST showed a moderate negative correlation with SVA and a significant negative correlation with T1-SPI and a moderate negative correlation with T9-SPI (*r = *−0.426, *P = *0.005; *r = *−0.659, *P = *0.000; *r = *−0.498, *P = *0.001); T1-SPI showed a significant positive correlation with T9-SPI (*r = *0.759, *P = *0.000); T1 pelvic angle showed a significant positive correlation with LPA(*r = *0.609, *P = *0.000) (**Table 3).**

**Table 3:** Correlations among sagittal plane parameters in patients with AIS.

Classification	*r*	*p*
LL _VS_ TK	0.335	0.033
LL _VS_ SS	0.776	0.000
LL _VS_ PI	0.682	0.000
LL _VS_ SSA	0.762	0.000
PT _VS_ PI	0.370	0.017
PT _VS_ ST	0.335	0.032
PT _VS_ SPA	−0.537	0.000
PT _VS_ T1-SPI	−0.579	0.000
PT _VS_ T9-SPI	−0.639	0.000
SS _VS_ PI	0.731	0.000
SS _VS_ CCA	−0.312	0.047
SS _VS_ SSA	0.815	0.000
SS _VS_ T9-SPI	0.352	0.024
PI _VS_ SSA	0.664	0.000
PI _VS_ SPA	−0.417	0.007
SVA _VS_ ST	−0.426	0.022
SVA _VS_ T1-SPI	0.378	0.015
SVA _VS_ T9-SPI	0.316	0.044
ST _VS_ T1-SPI	−0.659	0.000
ST _VS_ T9-SPI	−0.498	0.001
T1-SPI _VS_ T9-SPI	0.759	0.000
TPA _VS_ LPA	0.609	0.000

### 3.5 Binary logistic regression analysis

Binary logistic regression analysis revealed that the SPA was statistically significant (*P = *0.007), with an odds ratio and 95% confidence interval of 1.568 (1.129–2.177). No statistical significance was found for the remaining parameters (*P > *0.05) (Table 4).

**Table 4:** Binary Logistic Regression Analysis of Sagittal Parameters.

Sagittal Parameters	β	SE	waldX^2^	OR	95%CI	*P*
TK(°)	0.130	0.067	3.820	1.139	1.000-1.297	0.051
LL(°)	−0.003	0.072	0.002	0.997	0.865-1.148	0.963
PT(°)	0.392	0.237	2.727	1.480	0.929-2.357	0.099
SS(°)	−0.031	0.136	0.052	0.969	0.743-1.265	0.819
PI(°)	−0.005	0.122	0.002	0.995	0.784-1.263	0.969
CCA(°)	0.029	0.040	0.527	1.029	0.952-1.113	0.468
TLK(°)	0.029	0.060	0.232	1.029	0.915-1.158	0.630
SVA(mm)	0.000	0.010	0.001	1.000	0.981-1.020	0.973
SSA(°)	0.043	0.096	0.201	1.044	0.865-1.259	0.654
ST(°)	0.320	0.186	2.961	1.377	0.957-1.982	0.085
SPA(°)	0.449	0.167	7.201	1.568	1.129-2.177	0.007
T1-SPI(°)	0.486	0.287	2.864	1.625	0.926-2.852	0.091
T9-SPI(°)	0.217	0.189	1.317	1.242	0.858-1.800	0.251
TPA(°)	0.171	0.116	2.163	1.186	0.945-1.490	0.141
LPA(°)	0.130	0.114	1.302	1.138	0.911-1.422	0.254

## Discussion

Idiopathic scoliosis can be divided into three peak periods of onset: infancy, juvenile, and adolescence; however, it primarily affects adolescents, with AIS accounting for 80% of idiopathic scoliosis cases [1,23]. Correcting sagittal plane deformities is an important surgical objective in patients with AIS, and there is a notable correlation between spinal coronal and sagittal plane parameters in patients with AIS. Preoperative assessments should determine the optimal relation between these two orientations to maintain overall spinal balance [24,25]. Quantifying spinal radiographic sagittal parameters helps in understanding individual differences and the relation between spinal morphology and spinal diseases [26]. Therefore, this study compared the sagittal parameters between healthy adolescents and patients with AIS to enhance our understanding of sagittal parameter differences in patients with AIS, contributing to disease awareness. However, because of the complex structure of the spine and diversity of conditions, corrections should not consider only a single plane or segment. Comprehensive preoperative patient assessment is necessary to develop a reasonable surgical plan. Measuring trunk imbalance severity based on imaging is crucial for the selection and evaluation of treatment plans. Currently, pelvic parameters, CCA, TLK, TK, LL, and SVA are key components in studying the sagittal balance of the spine and pelvis [9,17]. Numerous new measurement parameters have also been applied to evaluate sagittal spine-pelvis balance, either as entirely new metrics or as combinations of existing parameters. For instance, T1-SPI, T9-SPI, TPA, and LPA are indices used to assess overall sagittal spine-pelvis balance [18]. Additionally, parameters like ST, SSA, and SPA not only reflect overall sagittal balance but also incorporate spinal length into consideration, highlighting the individuality of the parameters [19,20]. This study utilized a multicenter, multidimensional, and multiparameter assessment approach to quantify the sagittal characteristics of patients with AIS, comparing the differences in parameters between healthy adolescents and patients with AIS and among patients with AIS of different sexes, to provide reliable sagittal reference opinions for preoperative evaluation.

This study employs logistic regression analysis to explore the relationship between quantified parameters and the overall spine, aiming to assess the impact factors of the disease [8,27]. The results showed an increase in the SPA among patients with AIS (odds ratio = 1.568, 95% confidence interval = 1.129–2.177, *P = *0.007). SPA was moderately negatively correlated with PI and PT (*r = *−0.417, *P = *0.007; *r = *−0.537, *P = *0.000), indicating that an increase in SPA in patients with AIS leads to a decrease in PI and PT, mitigating the impact of spinal curvature on the overall spinal-pelvic balance. This aids in maintaining the overall balance and upright posture of the body. Therefore, preoperative planning should focus on assessing the relation between the sagittal position of the spine and the pelvis.

Sex differences are a significant factor in the study of AIS, with women having a higher incidence rate and severity of the disease than men [24,28]. Spinal deformities lead to an increase in inclination, with the SSA, ST, and SPA effectively reflecting spinal inclination [19,20,29], as these indicators can be linked to the lower limbs, collectively reflecting the overall sagittal compensatory status of the spine. Research by Janssen M et al. [30] in 60 asymptomatic youths found that women had a higher vertebral tilt in the T1-L2 segments than men. Hu P et al. [31] compared sagittal plane parameters between normal men and women, finding that normal women have higher LL, PI, SSA, and SVA than that of normal men. Wang W [19] found that normal women had higher SSA and ST values than those of normal men. This study’s results on sex differences in patients with AIS showed that TK, SS, SPA, T1-SPI, and T9-SPI were higher in men than in women (*P = *0.006; *P = *0.022; *P = *0.002; *P = *0.011; *P = *0.023); ST was higher in women than in men (*P = *0.034), indicating that women with AIS have a greater degree of spinal inclination, and preoperative assessments should particularly focus on evaluating the spinal inclination in female patients. T1-SPI and T9-SPI can reflect the sagittal balance status of the spine, aiding in understanding the inclination condition of the spine in patients with AIS. Guo J et al. [32] found that with disease progression in patients with AIS, PT, T1-SPI, and T9-SPI gradually decreased. In this study, the average values of PT, T1-SPI, and T9-SPI for healthy adolescents and patients with AIS were 10.22°, −3.04°, and −7.70°, and 8.62°, −2.61°, and −7.08°, respectively, indicating a decrease in PT, T1-SPI, and T9-SPI in patients with AIS; however, T1-SPI and T9-SPI tended to tilt forward. This compensatory mechanism may be related to the body’s gravity line passing through T1 and T9 [33], representing an adaptive change in the body to improve spinal alignment and reduce scoliotic curvature, thus aligning the spine and pelvis more closely to that of healthy adolescents.

Vertebral deformities and torsion can cause coronal displacement of the spine; in severe cases, significant changes in the sagittal structure may occur. There is a correlation between different diseased spinal segments [9,34]. Therefore, thoroughly studying the correlations among spinal sagittal parameters plays an important role in understanding the three-dimensional geometric characteristics of the spine and assessing the severity of conditions [35]. Spinopelvic parameters are key indicators of the alignment and balance of the spine and pelvis in the sagittal plane. Various causes leading to pelvic disorders, including an increase sacral slope, necessitate compensatory adjustments, such as an increase in LL and TK to maintain sagittal balance in the body [17]. Clément J L et al [36]. noted that LL in patients with AIS is influenced by TK and PI. Tanguay F et al [13]. highlighted significant correlations among sagittal lumbar-pelvic parameters in patients with AIS, including PI with SS and PT and LL with PI and SS, while PT and SS showed no correlation. Preoperative assessments should determine the optimal relation between the LL and pelvic morphology. Wu C et al. [23] found significant correlations between SS and LL, PI in the coronal and sagittal parameters of patients with AIS, with a moderate correlation between TK and LL. The results of this study are similar to those of previous research [13,17,23,36]; additionally, our study’s pelvic parameters conformed to the formula PI = PT + SS [23,37], consistent with findings from healthy adolescents (*t = *3.858, *P = *0.052) and patients with AIS (*t = *0.855, *P = *0.357) by Wu C [19] and Zhang R F[37]. Building on these studies, we conducted a multi-parameter correlation analysis of overall, regional, and local imbalances in the sagittal position of the spines of patients with AIS. SSA showed significant positive correlations with LL, SS, and PI (*r = *0.762, *P = *0.000; *r = *0.815, *P = *0.000; and *r = *0.664, *P = *0.000, respectively), indicating a connection between overall and local/regional imbalances. An overall imbalance may lead to local/regional imbalances and vice versa; however, the relations between the parameters are not merely mutually reinforcing. SPA was negatively correlated with PI and PT (*r = *−0.417, *P = *0.007; *r = *−0.537, *P = *0.000), suggesting that a decrease in PT (reduced PT) or a decrease in pelvic inclination (reduced PI) resulted in a further backward shift of the spine relative to the pelvis (increased SPA). This reflects an adaptive adjustment of the body to maintain spinopelvic balance, ensuring horizontal sight and spinal equilibrium.

This study has certain limitations. First, the sample size was relatively small, and further research with a larger sample size is warranted. Second, this study analyzed sagittal balance parameters without incorporating coronal and horizontal plane indicators. Future research should conduct correlation analyses of the measurement parameters across different planes. Finally, In future studies, while expanding the research scope, it is important to incorporate the Lenke classification, gait analysis, and other research to further elucidate the relationship between sagittal parameters and patient posture.

## Conclusion

AIS leads to an increase in SPA in patients, and preoperative evaluations should focus on the relation between the sagittal positions of the spine and pelvis. Sex differences in patients with AIS show that ST is higher in women than in men, and preoperative assessments should particularly focus on evaluating the spinal inclination in female patients.
